# Effects of the Order of Physical Exercises on Body Composition, Physical Fitness, and Cardiometabolic Risk in Adolescents Participating in an Interdisciplinary Program Focusing on the Treatment of Obesity

**DOI:** 10.3389/fphys.2019.01013

**Published:** 2019-08-06

**Authors:** Braulio Henrique Magnani Branco, Débora Valladares, Fabiano Mendes de Oliveira, Isabelle Zanquetta Carvalho, Déborah Cristina Marques, Andressa Alves Coelho, Leonardo Pestillo de Oliveira, Sônia Maria Marques Gomes Bertolini

**Affiliations:** ^1^Research Group in Physical Education, Physiotherapy, Sports, Nutrition and Performance of the University Center of Maringá (GEFFEND/UniCesumar), Maringa, Brazil; ^2^Postgraduate Program in Health Promotion (PPGPS/UniCesumar), Maringa, Brazil

**Keywords:** adolescent health, cardiometabolic risk in childhood, childhood obesity, exercise physiology, health promotion, interdisciplinary research

## Abstract

The main objective of this study was to investigate the effects of the order of physical exercises on body composition, physical fitness, and cardiometabolic risk in adolescents participating in an interdisciplinary program focusing on the treatment of obesity. The final 12-week analyses involved 33 female adolescents who were split into two groups of concurrent training (CT): resistance plus aerobic training and aerobic plus resistance training, with equalization performed in all physical exercises. The only difference between the two groups was the order in which the exercises were performed. The results showed reductions in fat mass, body fat, and waist circumference, as well as increases in musculoskeletal mass and resting metabolic rate (*p* < 0.05) following the multiprofessional intervention period. However, no significant differences were observed in regard to body mass, body mass index, neck circumference, or arm circumference (*p* > 0.05). Maximal isometric strength and maximal oxygen consumption showed significant increases after the intervention period (*p* < 0.05). There were reductions in insulin, HOMA-IR, total cholesterol, triglycerides, and low-density lipoproteins (*p* < 0.05), and an interaction within the resistance plus aerobic training group showed lower values for triglycerides when compared to itself (*p* = 0.002). No difference was found in fasting glycemia for either group (*p* > 0.05). It is worth noting that the equalization training variables presented no differences between the two groups (*p* > 0.05). Based on these results, both CT methods were found to be effective in promoting health parameters in overweight and obese female adolescents, and triglyceride values decreased more in the resistance plus aerobic group. Future studies with larger samples and feeding control should be conducted to confirm or refute our findings.

## Introduction

Obesity is substantially associated with increased caloric intake, low levels of physical activity, and reduced energy expenditure (Blüher, 2019). The literature has indicated that, by 2015, more than 100 million children and adolescents worldwide were classified as obese in both developed and developing countries (Lee and Yoon, 2018). Furthermore, obesity in adolescence exponentially increases the risks of developing other diseases, including diabetes mellitus and hypertension and other cardiovascular diseases in adulthood (Ho et al., 2019).

Currently, the scientific literature still lacks controlled studies seeking to identify the effects of physical exercise (PE) intensity on weight loss in adolescents since most studies present a low volume of interventions (Stoner et al., 2019). Therefore, consensus among researchers regarding the most efficient physical training method for reducing body fat (BF) in adolescents is lacking. Among the recommended strategies, multiprofessional intervention has been noted for the treatment of obesity (Branco et al., 2018).

In addition, Petridou et al. (2019) indicated that resistance exercises (RE) are relevant for the treatment of obesity due to increased resting metabolic rate (RMR) and fat-free mass, which consequently increase the day-to-day energy expenditure. The same authors suggested that aerobic exercises (AE) should be incorporated in weight-loss programs since, depending on volume and intensity, the energy expenditure during the session may be greater than that in resistance training (RT), especially when the resting intervals are very long.

Recently, Branco et al. (2018) compared the effects of two different concurrent training methods on body composition, physical fitness, and cardiometabolic risk of obese adolescents. The two physical-training protocols were similar in volume, intensity, and types of exercises. The only difference was the means by which the exercises were performed, i.e., one group used body weight and the other group used machines, and when possible, the exercises were equalized by the primary muscle groups involved in achieving the activity. The results of all analyzed variables were similar since the volume, intensity, and exercises performed were equalized.

Consistent with Kang and Ratamess (2014) regarding the choice of the protocol used to conduct concurrent training, AE or RE at the beginning or end of the session will depend on the first objective of physical training for athletes of different modalities. However, this condition is recommended for athletes but not for sedentary individuals or for the treatment of certain chronic diseases. The same authors noted that the choice of the order of PE within the same training session can be organized according to the specificity of the modality, i.e., for athletes with aerobic resistance modalities, performing aerobic training first to increase the quality of their training is recommended. However, for athletes seeking muscular hypertrophy, it is recommended that RE be performed at the beginning of the training session for the same reason mentioned above. The World Health Organization [WHO], (2010) suggested that children and adolescents need to improve cardiorespiratory fitness and muscular endurance and strength. Thus, a strategy that may be used is concurrent training (Coffey and Hawley, 2017). Furthermore, Vilaça et al. (2011) emphasized that a combination of AE and RE (regardless of order) is beneficial for reducing BF and increasing energy expenditure during and after PE sessions.

To the best of the author’s knowledge, the chronic physiological and metabolic responses under the order of concurrent training in obese sedentary adolescents are unknown. Regardless of the order of the execution of PE, there is no consensus or indication regarding which model/order of PE should be followed in order to enhance improvement in components related to physical fitness in terms of health. Considering these aspects, the identification of physiological and metabolic responses during respective intervention models could direct the interventions and even prevent paradigms or concepts based on the common sense of health professionals, especially those involved in prescribing PE. Therefore, the aim of the present study was to investigate the effects of the order of physical exercises on body composition, physical fitness, and cardiometabolic risk in female adolescents participating in an interdisciplinary program focusing on the treatment of obesity. Regarding the hypothesis, it is believed that the responses observed in the different parameters analyzed will be similar since only the order of the exercises was changed.

## Materials and Methods

### Participants

**FIGURE 1:**
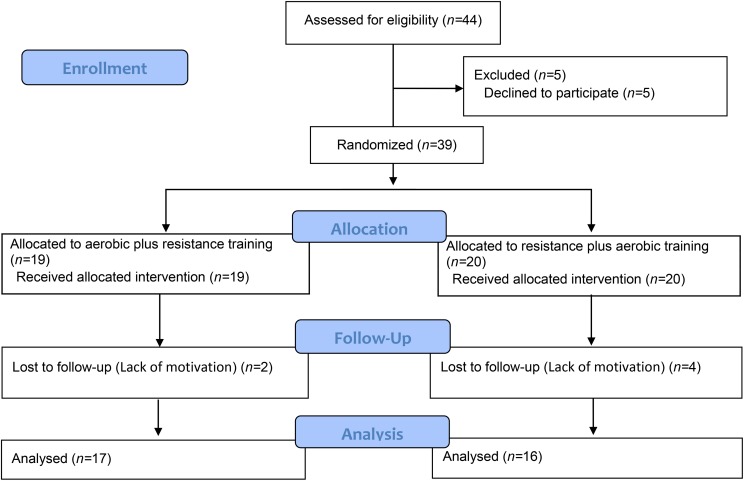
The flowchart of the present study.

A total of 44 female adolescents were recruited according to the following established inclusion criteria: (1) age between 13 and 17 years; (2) overweight or obesity according to the cutoff ranges established by Cole and Lobstein (2012); and (3) availability to participate in multiprofessional interventions 3× per week during the evening for a period of 12 weeks. The exclusion criteria were as follows: (1) participating in sports practices systematized outside of school, (2) orthopedic, cardiovascular, or cognitive deficits that impede practicing PE, (3) some type of restrictive diet, i.e., low-carb, low-fat or hypocaloric, (4) the use of a psychotropic or appetite-regulating medicine, and (5) completion of less than 75% of the multiprofessional interventions during the 12-week intervention. It is emphasized that the adolescents were encouraged to be more physically active during the intervention (outside the university setting). In relation to the variables, a sample size with nine participants in each group intervention was enough to detect eventual changes in dependent variables, with standard deviation smaller when compared to previous study, with 80% confidence (Branco et al., 2018). The details of the process are provided in Figure 1, which depicts the flowchart for the present study.

### Ethics Statement

This study was approved by the Committee for Ethics in Research of the University Center of Maringá (UniCesumar) through the opinion n° 2,505,200/2018. The research followed all recommendations proposed by resolution 466/2012 of the Ministry of Health of the Brazilian Government and the Declaration of Helsinki. All parents or guardians of the adolescents were informed in detail about the purposes of the study and subsequently signed an informed consent form. In addition, the adolescents signed a consent term agreeing to participate.

### Study Design

This study adopted an experimental, longitudinal, parallel-group, and repeated-measures design. Previously, the adolescents were randomly allocated to one of the following two experimental groups: G1 = aerobic plus resistance group or G2 = resistance plus aerobic group. The only distinction between the groups was the order of performing the activities, i.e., G1 performed AE followed by RE, whereas G2 performed the activities in the inverse order, i.e., RE followed by AE. The details of the aerobic and resistance exercises in regard of volume and intensity are presented in the discussion below. The participants performed the interventions three times a week for 12 weeks guided by a team of professionals in physical education, nutrition, and psychology. PE was performed 3× per week divided into training A and B, as theoretical and practical classes related to nutrition were conducted 2× per week, and cognitive-behavioral therapy was provided 1× per week over the course of the 12-week intervention. On the first day of evaluations, the adolescents underwent a medical consultation involving the following: (a) anamnesis, (b) measurement of resting blood pressure, (c) pulmonary and cardiac auscultation, (d) completion of the International Physical Activity Questionnaire (IPAQ), and (e) release for venous blood collection. On the second day, the following were performed: (a) venous blood collection following ∼ 12 h of fasting, (b) body composition assessment, (c) anthropometry, and (d) explanation and collection of food records. On the third day, physical tests were performed in the following order: (a) maximal isometric handgrip test (MIHT), (b) maximal isometric lumbar-traction strength (MILTS), (c) maximal isometric lower-body strength (MILBT) and (d) aerobic fitness. The 3 days of evaluations were separated by a 48-h interval. Considering that the interventions were performed on Mondays, Wednesdays, and Fridays, the evaluations began on the same days, and re-evaluations were conducted after the weekend. The girls rested for 2 days, and the process followed the same days of evaluation in the beginning of this study.

### Exercise Training Protocol

The training sessions were divided into two mesocycles performed in circuit form with series A and B. The two series were performed alternately to promote the physiological recovery of the muscle groups previously worked. The training schedule was divided into two mesocycles of 6 weeks duration each. Regarding the exercises, we chose to perform PE without repetition counting and to control the exercises by timing the activities. During the first mesocycle, the effort: pause ratio was 30″ by 30″, while for the second mesocycle, the effort: pause ratio was 40″ by 20″ using a 1:1 ratio between the concentric and eccentric phases. In addition, before the session, the adolescents performed a 10-min warm-up as follows: light walking and stretching of the main muscle groups (with low muscle tension). To periodize the training, promote anatomical adaptation, and reduce any lesions, the intensity of the sessions was controlled by the Borg (1982), which includes values of 6 arbitrary units (a.u.) up to 20 a.u., i.e., from very easy to exhausting. Table 1 shows the AE performed by both experimental groups.

**TABLE 1:** Systematization of aerobic training during 12 weeks of intervention.

**Order**	**Exercises**	**Effort time**	**Type of exercise**	**Intensity**
1	Light jogging	4 min	Continuous	9–10 a.u.
2	Running	4 min	Continuous	13–14 a.u.
3	Sprints during 40 m and active self-selected recovery for 20 m	8 min	HIIE	*All-out* mode
4	Group activities	4 min	Interval activities	Self-selected
	Total per session	20 min		

*Group activities involved collective games and recreation; HIIE, high-intensity interval exercise; a.u., arbitrary units of Borg scale (6–20).*

Table 2 shows the intensity of the resistance efforts during the 12-week intervention.

**TABLE 2:** Intensity of the resisted efforts during the 12 weeks of intervention.

**Week**	**Intensity**	**Series**	**Time per session**
1st and 2nd	10–12 a.u.	2×	18 min + 3 min of rest + 5 min of slowdown = 26 min
3rd and 4th	10–12 a.u.	3×	27 min + 6 min of rest (3 min per series) + 5 min of slowdown = 38 min
5th and 6th	12–14 a.u.	2×	18 min + 3 min of rest + 5 min of slowdown = 26 min
7th and 8th	12–14 a.u	3×	27 min + 6 min of rest (3 min per series) + 5 min of slowdown = 38 min
9th and 10th	15–17 a.u.	2×	18 min + 3 min of rest + 5 min of slowdown = 26 min
11th and 12th	15–17 a.u.	3×	27 min + 6 min of rest (3 min per series) + 5 min of slowdown = 38 min

*After each series that was performed in a circuit format, the adolescents rested for 3 min and then the exercises continued until the series were completed, according to the training weeks. In slowdown, it was performed low intensity aerobic exercises and light intensity stretching exercises were used for the most used muscle groups during the session; a.u., arbitrary units of Borg scale (6–20).*

Table 3 presents the training program groups A and B performed during the first and second RT mesocycles.

**TABLE 3:** First and second mesocycle of resistance training of both experimental groups.

**Order**	**Training program A**	**Order**	**Training program B**
**First mesocycle of RT (effort: pause ratio 30″ per 30″)**			
1	Push-ups (on knees)	1	Suspended row
2	Half squat	2	Hip bridge
3	Plank	3	Crunch abdomen
4	Medicine ball chest throw (with partner)	4	Pulling tire with rope
5	Box march	5	Standing calf raise
6	Medicine ball alternating side rotation throw	6	Crunch abdomen throwing medicine ball
7	Medicine ball overhead throw	7	Upright Row with dumbbells
8	Half squat in isometric position	8	Stiff with dumbbells
9	Twisting Sit-up	9	Abdomen with Swiss ball
**Second mesocycle of RT (effort: pause ratio 40″ per 20″)**			
1	Push-ups (on knees) using a step (progression of the previous physical exercise)	1	Suspended row - neutral grip
2	Thruster with medicine ball	2	Hip bridge
3	Plank	3	Twist abdomen with medicine ball
4	Throw wall-ball on the wall	4	Rope tsunami + half isometric squat
5	Low skipping	5	Agility ladder: lateral displacement exercises
6	Medicine ball alternating side rotation throw	6	Crunch abdomen throwing medicine ball
7	Half squat in isometric position with Swiss ball between the legs	7	Suspended row - pronated grip
8	High skipping	8	Flexion and knee extension with Swiss ball
9	Plank changing arms and legs	9	Abdomen with Swiss ball

*RT, resistance training; each mesocycle presented 6 weeks of duration.*

### Training Monitoring

To monitor the physical training sessions, several non-invasive and practical instruments were used by the physical trainer. Prior to each training session, the rating of the perceived recovery (RPR) scale as proposed by Laurent et al. (2011), the rating of perceived exertion (RPE session) as proposed by Foster et al. (2001), and the internal training load (ITL) were assessed according to the considerations proposed by Nakamura et al. (2010). The RPR indicates the recovery status and is used before each training session. The scale comprises numerical values ranging from 0 a.u. (very poor recovery/extremely tired) to 10 a.u. (very good recovery/extremely well-disposed). The RPE session was applied 30 min after the end of the training sessions. This scale comprises values ranging from 0 a.u. (extremely light effort) to 10 a.u. (extremely heavy stress). The ITL was calculated by multiplying the value of the RPE by the duration of the training session in minutes. The girls were familiarized with the scales before and during interventions. The scales were administered to the girls, and explanations were provided during the theoretical classes and before the PE sessions.

### IPAQ Questionnaire

The IPAQ questionnaire adapted for adolescents was used to identify the levels of physical activity before and after the interdisciplinary interventions and to determine whether the adolescents had already engaged in other moderate/intense physical activities before starting the program (Guedes et al., 2005).

### Nutrition Intervention

The nutritional interventions aimed to guide the adolescents in nutritional aspects such as: (a) introduction to healthy eating: food manufacturers, regulators, energy, and the food pyramid, (b) frequency and portioning of food, (c) soluble and insoluble fibers, (d) the importance of fiber consumption, (e) a list of food replacements, according to the food groups, (f) pre- and post-physical activity feeding, (g) minimally processed, processed, and ultra-processed foods, (h) amounts of sugar, salt, and fat in foods, (i) how to read and interpret food labels, (j) diet and “light” foods, (k) eating disorders, (l) the dangers of “fad diets”, and (m) ingesting food for health and quality of life using food re-education as an instrument. Those responsible were advised to note, in specific forms, all foods and beverages consumed throughout the day, and also to record the size of portions consumed; the name of the preparations, their ingredients, and the method preparation. They were also instructed to note details such as the addition of salt, sugar, oil, sauces, etc., and whether the food or drink consumed was regular, diet, or light. For the best estimation of the portion size, they used home measurements (Slater et al., 2002). The classes, which were conducted by means of theoretical activities and practical dialogues, lasted approximately 1 h and took place 2× per week. A 3-day food recall was applied on two non-consecutive days of the week and on 1 day at the end of the week. All data were tabulated using the program Avanutri (Avanutri Equipamentos de Avaliação Ltda, Três Rios, Brazil). Another point that needs to be highlighted was the absence of feeding control. It was suggested that the teenagers change their dietary habits according to theoretical and practical classes on nutrition and psychological interventions. However, a control of percentages of macronutrient ingestion or the quality of the food consumed was not performed during this study. Finally, the kilocalories/day ingested by the adolescents were calculated, and the values pre- and post-intervention were compared. The intervention model and food-recall analysis procedures were the same as those performed in the study conducted by Branco et al. (2018).

### Psychological Intervention

The primary themes of the psychological intervention were cognitive and behavioral strategies used to improve the quality of food consumption among the adolescents, including self-monitoring, control of anxiety, negative feelings, and health education. The activities also aimed to reduce negative emotions regarding the participants’ relationships with food consumption and strategies used to improve the assessment of body self-image. Psychological intervention was divided into four large groups of subjects, and a cognitive-behavioral methodology in a group setting was used once a week for 1 h.

#### Eating Habits

Discussions about food re-education explored the eating habits of the participants and their families.

#### Self-Monitoring

Self-monitoring is an important variable to be discussed in the behavioral treatment of obesity. The meetings held with this theme were conducted with the following characteristics: group activities to increase mutual knowledge and foster interaction among the participants, discussions about the advantages of having a healthy lifestyle for the whole family and alluding to the practice of exercise and its advantages.

#### Control of Anxiety and Negative Feelings

The purpose was to promote reflection on anxiety and to explore the fears, difficulties, and expectations of the participants. The purpose of the meetings with this theme was to discuss issues related to the acquisition of behaviors considered healthy and to reduce the negative feelings related to anxiety, as well as to promote the participants’ self-knowledge so that they could reflect on positive and negative thoughts that permeate their daily lives.

#### Health Education

The purpose of the meetings was to raise awareness about issues related to the reality of the group, for example, to demystify beliefs, prejudices, and stereotypes associated with obesity. The interventions followed the model proposed by Branco et al. (2018).

### Medical Consultation

Prior to all activities, the adolescents consulted with a medical team. They were interviewed following a structured anamnesis to investigate their clinical history, previous and current pathologies, and their use of medications. In addition, pulmonary and cardiac auscultation and blood pressure measurements were performed according to the 7th Brazilian Guideline of Arterial Hypertension (Malachias et al., 2016).

### Biochemical and Hormonal Tests

The adolescents arrived at the biochemistry laboratory of the teaching institution after fasting for approximately 12 h. After local asepsis of the arm, a puncture was performed on the antecubital veins of the evaluated adolescents. All analyses were performed by a biomedical team blinded to the details of the interventions, i.e., the team did not have access to the intervention model realized by the adolescents during the 12-week intervention. All analyses were performed in triplicate using Siemens reagents (Frimley, Camberley, United Kingdom) in accordance with the manufacturer’s specifications. Subsequently, the samples were centrifuged at 3,600 rpm for 11 min at a controlled temperature (24°C) for the separation of the serum and plasma. Vacuum tubes (Becton Dickinson Vacutainer^®^, Plymouth, United Kingdom) were used for all collections, namely a tube with potassium fluoride and ethylenediaminetetraacetic acid (EDTA) was used for the analysis of the fasting plasma glucose level, and a clot activator tube (silica) was used for the analysis of the insulin, total cholesterol (TC), high-density lipoprotein (HDL-c), low-density lipoprotein (LDL-c), and triglyceride (TG) serum levels. For the biochemical analyses, Siemens equipment (Advia 1800 Chemistry Analyzer^®^, Siemens Healthcare Diagnostics, Illinois, United States) was used. The formula HOMA-IR = fasting glucose (nmol/L) ^∗^fasting insulin test (HOMA-IR) was used to calculate the homeostasis model assessment (HOMA) to evaluate insulin resistance [μU/mL)/22.5] according to Matthews et al. (1985).

### Body Composition Assessment

Electrical bioimpedance (BIA) was conducted using the eight-tailed InBody four-quadrupole multifrequencial apparatus (model 570^®^ Body Composition Analyzers, Seoul, South Korea). On the first day of evaluations, all participants received an informative document containing the protocol used to perform the assessment, including (a) 12-h fasting, (b) no moderate or vigorous physical exercise within the 24 h preceding the test, (c) urination and evacuation for the evaluation, (d) the absence of metallic objects during the evaluation, (e) postponement of the assessment in menstruating participants until after their period, (f) at least 8 h of sleep, and (g) no ingestion of caffeinated beverages or foods during the 12 h prior to the measurement (Heyward, 2001; Branco et al., 2018b). The following BIA variables were evaluated: body weight, body mass index (BMI), musculoskeletal mass (MME), fat mass (FM), %BF, and RMR.

### Anthropometry

Stature was measured by an electronic scale with a stadiometer (Filizola^®^, São Paulo, Brazil) with a capacity of 200 kg, with a measuring capacity of 2 m and accuracy of 0.1 cm. Waist (WC), relaxed right arm (AC), and neck (NC) circumferences were measured following the protocol proposed by Heyward (2001). An extensible measuring tape of the WISO brand (model T87-2^®^, Florianopolis, Santa Catarina, Brazil) was used with a measuring capacity of 2 m and precision within 0.1 cm.

### Physical Assessment

Before the measurements were obtained, all adolescents were familiarized with the MIHS, MILTS, MILBS and aerobic fitness tests (Branco et al., 2018a).

#### MIHS

A Jamar dynamometer (Asimow Engineering, Los Angeles, CA, United States) was used to evaluate MIHS. The teenager remained standing while holding the device close to her body with her arm extended with a neutral grip. Following a signal by the evaluator, the adolescent squeezed the dynamometer as hard as possible while maintaining the isometric contraction for 3 to 5 s. The measurement was performed with both hands. It was used the sum of right and left hand (sum of maximum isometric handgrip strength of right and left hand – SMIHS, in the statistical analysis), for After three attempts on each side, the maximum isometric strength of the handgrip of the right and left hands was recorded. A 60-s interval separated each attempt (Branco et al., 2018a).

#### MILTS

A Kratos dynamometer (Kratos Industrial Equipment, Model DS^®^, São Paulo, Brazil) was used to evaluate MILTS. The teenager walked with both feet on the device with her knees extended, trunk flexed approximately 120°, head and neck aligned with the trunk, and fingers (holding the bar) positioned in front of the patella bone. The maximum contraction over 3 to 5 s was recorded, and the highest value of the measurement after three attempts with 60 s of rest between attempts was recorded (Branco et al., 2018a).

#### MILBS

To evaluate the maximal isometric strength of the lower limbs, a Kratos dynamometer also used (the same equipment of evaluation of MILTS). The teenager stood on a platform with her knees bent at approximately 120° and trunk upright while holding the traction bar lined up in the inguinal fold. Following the evaluator’s signal, the adolescent performed the maximum isometric contraction for 3 to 5 s, and the highest measured value after three attempts with 60-s resting intervals was recorded (Branco et al., 2018a).

#### Aerobic Fitness

To measure aerobic fitness, the maximal oxygen consumption (VO_2_max) test, which was proposed by Léger and Lambert (1982), was used. This test has 21 stages; the initial velocity is 8.5 km/h, which increases by 0.5 km/h per stage. The VO_2_max was calculated by the following equation:

VO⁢max2=31.025+(3.288*⁢X)-(3.248*⁢A)+(0.1536*⁢A*⁢X)

where X = velocity in the stage reached, and A = age in years.

### Statistical Analysis

Previously, the normality of the data was tested using the Shapiro–Wilk test. Similarly, the homogeneity of the data was tested by Levene’s test. After confirming normality and homogeneity, the data were expressed as the mean and (±) standard deviation. Mauchly’s test of sphericity was used to test as well as the Greenhouse-Geisser correction, if necessary. A two-way mixed-measures analysis of variance (ANOVA) was used to compare the groups and phases (pre- and post-intervention). Bonferroni *post hoc* test was used when a significant difference was found. A significance level of 5% was adopted for all analyses. In addition, the effect size was calculated using Cohen’s d (1992) as follows: 0.20 (*small effect*), 0.50 (*moderate effect*), and 0.80 (*large effect*). Furthermore, the partial eta-squared: η*p*^2^ was calculated with the following classification: 0.10 (*small effect*), 0.25 (*moderate effect*), and 0.40 (*large effect*), according to Cohen (1992). All statistical analyses were performed using the statistical package SPSS 22.0 (IBM, Inc., United States).

## Results

Information regarding age, body weight, and stature is provided in Table 4.

**TABLE 4:** Body composition and anthropometric measurements of female of adolescents participating in the study.

**Variables**	**Aerobic plus resistance training group**			**Resistance plus aerobic training group**		
	**Pre**	**Post**	***Cohen’s d***	**Pre**	**Post**	***Cohen’s d***
Age (years old)	13.4 ± 2.0	13.9 ± 2.1	0.2	13.1 ± 3.0	13.1 ± 2.5	0.0
Body mass (kg)	90.8 ± 23.7	87.5 ± 19.3	–0.1	86.5 ± 21.1	85.8 ± 21.0	–0.0
Stature (cm)	162.0 ± 8.7	164.4 ± 9.6	0.3	161.1 ± 11.4	161.2 ± 10.5	0.0
BMI (kg/m^2^)	34.1 ± 7.1	32.2 ± 5.8	–0.3	33.6 ± 6.2	32.1 ± 5.4	–0.2
MME (kg)^∗^	27.6 ± 6.6	28.3 ± 6.6	0.1	27.0 ± 6.6	27.3 ± 7.0	0.0
FM (kg) ^∗^	41.7 ± 14.9	36.5 ± 11.9	–0.3	40.9 ± 13.8	36.5 ± 11.8	–0.3
BF (%) ^∗^	44.7 ± 6.4	41.3 ± 6.8	–0.5	44.6 ± 7.4	41.9 ± 6.8	–0.4
RMR (Kcal) ^∗^	1414.3 ± 216.1	1470.6 ± 237.6	0.3	1427.8 ± 241.3	1434.2 ± 235.6	0.0
WC (cm)^∗^	95.6 ± 13.1	91.7 ± 10.4	–0.3	93.6 ± 12.9	92.2 ± 11.3	–0.1
NC (cm)	35.9 ± 3.4	35.8 ± 3.0	0.0	36.2 ± 3.4	36.1 ± 3.0	0.0
AC (cm)	34.7 ± 4.5	34.7 ± 3.8	0.0	35.1 ± 5.2	35.5 ± 5.3	0.1

*Data are expressed as mean and (±) standard deviation; BMI, body mass index; MME, musculoskeletal mass; FM, fat mass; BF, percentage of body fat; RMR, resting metabolic rate; WC, waist circumference; NC, neck circumference; AC, relaxed right arm circumference; ^∗^time effect (*p <* 0.05).*

### IPAQ Responses

For the IPAQ, no significant differences were observed in the level of physical activity among the adolescents after the 12-week multiprofessional intervention (*p* > 0.05). The only difference observed was an increase in the level of physical activity performed on Mondays, Wednesdays, and Fridays. However, this result was expected since the adolescents participated in the activities 3× per week during the 12-week intervention.

### Training Responses

For the RPE, ITL, and RPR, no significant differences were identified during the same microcycles and mesocycles of training performed by the adolescents (*p* > 0.05). These responses suggest that both training groups were similarly equalized in recovery, volume, and intensity.

### Food Recall

For the caloric intake in kcal, no significant differences were observed before and after the 12-week intervention (*p* > 0.05). These responses suggest that the 12-week nutritional intervention is incipient for promoting significant changes in caloric intake.

Table 5 presents the body composition and anthropometric measurements of the adolescents participating in the interdisciplinary project for the treatment of obesity before and after the 12-week intervention.

**TABLE 5:** Physical tests of female of adolescents participating in the study.

**Variables**	**Aerobic plus resistance training group**			**Resistance plus aerobic training group**		
	**Pre**	**Post**	***Cohen’s d***	**Pre**	**Post**	***Cohen’s d***
SMIHS (kg) ^∗^	51.4 ± 13.2	55.0 ± 14.0	0.3	51.6 ± 16.5	53.1 ± 18.8	0.1
MILTS (kg) ^∗^	59.5 ± 14.5	64.1 ± 17.3	0.3	56.4 ± 24.5	65.0 ± 24.1	0.4
MILBS (kg) ^∗^	59.9 ± 15.6	70.8 ± 22.3	0.7	57.8 ± 28.2	75.2 ± 31.1	0.6
VO_2_max (mL/kg/min) ^∗^	35.4 ± 4.2	43.7 ± 6.6	2.0	37.9 ± 5.1	46.0 ± 8.4	1.6

*Data are expressed as mean and (±) standard deviation; SMIHS, sum of maximum isometric handgrip strength of right and left hand; MILTS, maximum isometric lumbar-traction strength; MILBS, maximum isometric lower-body strength; VO_2_max, maximal oxygen consumption; ^∗^time effect (*p <* 0.05).*

Age, body weight, stature, BMI, CN, and AC did not show group, time or interaction effects. However, specifically regarding MME [*F*_(1,31)_ = 5.71, *p* = 0.02, η*p*^2^ = 0.15, small] and RMR [*F*_(1,31)_ = 5.06, *p* = 0.03, η*p*^2^ = 0.14, small] a time effect was identified, with higher values after the intervention period when compared to the pre-intervention values, while FM [*F*_(1,31)_ = 7.79, *p* = 0.008, η*p*^2^ = 0.20, small],%BF [*F*_(1,31)_ = 19.43, *p* = 0.001, η*p*^2^ = 0.38, medium] and WC [*F*_(1,31)_ = 11.28, *p* = 0.002, η*p*^2^ = 0.28, medium] showed significantly lower values after the intervention period.

Table 5 shows the physical tests performed by the adolescents participating in the interdisciplinary project for the treatment of obesity before and after the 12-week intervention.

For MIHS [*F*_(__1__,__31__)_ = 4.13, *p* = 0.05, η*p*^2^ = 0.11, *small*], MILTS [*F*_(__1__,__31__)_ = 6.18, *p* = 0.01, η*p*^2^ = 0.16, *small*], MILBS [*F*_(__1__,__31__)_ = 13.49, *p* = 0.0008, η*p*^2^ = 0.30, *medium*] and VO_2_max [*F*_(__1__,__31__)_ = 116.35, *p* < 0.001, η*p*^2^ = 0.79, *large*] a time effect was identified, with higher values after the intervention period when compared to the pre-intervention values.

Table 6 shows the biochemical examination results of the adolescents participating in the interdisciplinary project for the treatment of obesity before and after the 12-week intervention.

**TABLE 6:** Hormonal and biochemical tests of female of adolescents participating in the study.

**Variables**	**Aerobic plus resistance training group**			**Resistance plus aerobic training group**		
	**Pre**	**Post**	***Cohen’s d***	**Pre**	**Post**	***Cohen’s d***
Fasting glycemia (mg/dL)	82.6 ± 4.4	82.0 ± 3.9	−0.1	81.7 ± 6.2	82.0 ± 8.8	0.0
Insulin (μ/mL)^∗^	13.7 ± 4.2	11.2 ± 3.4	−0.6	14.0 ± 6.7	11.3 ± 5.5	−0.4
HOMA-IR ^∗^	2.84 ± 0.94	2.28 ± 0.71	−0.6	2.93 ± 1.35	2.34 ± 1.38	−0.4
Triglycerides (mg/dL)^∗^	96.5 ± 30.2	86.9 ± 35.9	−0.3	113.9 ± 38.2	76.1 ± 27.2^#^	−1.0
Total cholesterol (mg/dL)^∗^	169.6 ± 25.5	150.3 ± 25.0	−0.8	171.9 ± 23.0	144.1 ± 30.5	−1.2
LDL-c (mg/dL)^∗^	100.3 ± 23.1	90.1 ± 17.4	−0.4	99.9 ± 20.8	88.3 ± 22.4	−0.6
HDL-c (mg/dL)^∗^	42.8 ± 6.9	50.1 ± 8.6	1.1	40.7 ± 9.6	48.6 ± 9.5	0.8

*Data are expressed as mean and (±) standard deviation; HOMA-IR, homeostatic model assessment; LDL-c, low density lipoprotein; HDL-c, high density lipoprotein; ^∗^time effect (*p <* 0.05); ^#^interaction with lower values at the post-intervention for the same experimental group (*p =* 0.001).*

Fasting glycemia levels, did not show group, time or interaction effects. Regarding insulin [*F*_(__1__,__25__)_ = 9.67, *p* = 0.004, η*p*^2^ = 0.28, *medium*], HOMA-IR, [*F*_(__1__,__25__)_ = 6.44, p = 0.01, η*p*^2^ = 0.22, *small*], TG [*F*_(__1__,__25__)_ = 12.12, *p* = 0.001, η*p*^2^ = 0.33, *medium*], TC [*F*_(__1__,__25__)_ = 22.59, *p* = 0.000, η*p*^2^ = 0.47, *large*] and LDL-c [*F*_(__1__,__24__)_ = 1.56, *p* = 0.003, η*p*^2^ = 0.06, *small*] a time effect was identified, with lower values after the intervention period when compared to the pre-intervention values. In addition, it was observed an interaction effect for TG [*F*_(__1__,__25__)_ = 4.29, *p* = 0.048, η*p*^2^ = 0.15, *small*] with lower values in the resistance plus aerobic group in post-intervention period when compared to the pre-intervention values (*p* = 0.002). Besides, for HDL-c [*F*_(__1__,__25__)_ = 29.43, *p* < 0.001, η*p*^2^ = 0.54, *large*] a time effect was identified, with higher values after the intervention period when compared to the pre-intervention values.

## Discussion

The main results of the present study indicated the following differences after 12 weeks in the two intervention groups: (a) increase in MME and RMR, (b) reduction in FM, %BF, and WC, (c) increase in MIHS, MILTS, and MILBT, (d) increase in VO_2_max, (e) reduction in insulin, HOMA-IR, TG, TC, and LDL-c levels, and (f) increase in HDL-c. In addition, an interaction was detected with lower values of TG in the resistance plus aerobic group after the intervention period. However, no differences were observed in fasting glycemia levels in the two experimental groups after the 12 weeks. As a result, the study’s hypothesis was confirmed.

In the condition that the concurrent protocols are somehow equalized, the training responses have a tendency to be similar (Branco et al., 2018). The findings of the present study indicate that MME increased significantly after 12 weeks of concurrent training concomitant with multiprofessional care in obese adolescents. Thus, muscle hypertrophy is intimately associated with the optimal volume and intensity of RE (Schoenfeld, 2010). A study conducted by Branco et al. (2018) did not identify muscle hypertrophy after 12 weeks of concurrent training in obese male adolescents. However, the volume of the resistance exercises conducted by Branco et al. (2018) was relatively lower than that in the present study. Regarding this discrepancy, Figueiredo et al. (2018) noted that volume can be considered the most effective variable in RE for muscle hypertrophy. However, in a letter to the editor, Souza et al. (2018) explained that RE is very complex, and due to methodological differences among scientific studies, determining the decisive variables for establishing what volume is critical for muscle hypertrophy is impossible. In light of these different findings and because scientific studies sometimes employ different intervention models with dissimilar body-composition analyses, it is believed that the key to the process of muscle hypertrophy is the optimal interdependence of volume and intensity based on the uniqueness of individuals. Thus, the differences observed between the study conducted by Branco et al. (2018) and the present study may be related to the higher volume of RE performed by the adolescents in this study.

Considering absolute FM and BF, significant reductions were identified after 12 weeks. However, it is suggested that the reduction in %BF is mainly related to the increase in energy expenditure resulting from the concurrent training practiced since no significant differences in caloric intake were detected during the 12-week period. In addition, no differences among IPAQ responses were identified during the 12-week intervention. Branco et al. (2018) identified no alterations in caloric intake inferred from food records for 2 days during the week and one weekend day and IPAQ responses over the course of 12-week multiprofessional interventions that focused on reducing body fat and cardiometabolic risk and improving the components of physical fitness related to health in adolescents. Despite the limitations of overestimation or underestimation during the completion of food records and the IPAQ, especially by adolescents, considering the above-mentioned aspects is recommended to determine whether the reduction in absolute and relative fat is closely linked to an increase in energy expenditure resulting from physical exercises performed daily for a duration of ∼60 min, reaching ∼180 min weekly. Thus, Donnelly et al. (2004) suggested that the addition of regular physical exercise over the medium and long term can result in additional energy expenditure (between 1000 and 2000 kcal per week depending on volume and intensity). However, regarding energy expended to be converted to reduce body fat, it cannot be compensated by increasing daily caloric consumption.

Well-controlled studies indicate that an elevation in RMR is closely related to increased levels of physical activity if dietary restrictions with a negative energy balance are not incorporated (Stiegler and Cunliffe, 2006). Thus, overall, the gain of MME directly affect elevation in the RMR (Stiegler and Cunliffe, 2006). However, studies in the literature note that MME accounts for only 20% of oxygen consumption at basal conditions (Brozek and Grande, 1955; Durnin and Passmore, 1967; Wahrlich and Anjos, 2001). Thus, importantly, the energy expenditure of the MME represents only 13 kcal/kg/day (McClave and Snider, 2001). Consequently, the increase in MME has a very low impact on the total energy expenditure/day of individuals. Therefore, it is essential to increase the performance of structured and unstructured physical activities and decrease the time spent performing low-intensity activities to increase energy expenditure throughout the day.

Regarding WC, significant reductions were identified after the multiprofessional intervention period. Branco et al. (2018) also identified a reduction in WC in overweight or obese adolescents after a 12-week intervention. Reduction in WC has a considerable impact on reduction in cardiometabolic risk (Freedman et al., 1999). As a result, despite the significant reduction in WC in the two intervention groups, the values are considered high (Patnaik et al., 2017). However, NC and AC did not exhibit significant differences, which is likely due to the short duration of the 12-week intervention. These two measures, NC and AC, have been associated with cardiometabolic risk (Jaiswal et al., 2017; Patnaik et al., 2017). Thus, both anthropometric measures can also be used in clinical practice for the evaluation of adolescents with overweight or obesity. From this perspective, Patnaik et al. (2017) identified that WC and NC are strongly correlated (*r* = 0.69), suggesting that both may be used to identify possible conditions related to cardiometabolic risk. In addition, Jaiswal et al. (2017) reported accuracy between 0.92 and 0.98 of the ROC curve of the association between arm circumference and overweight in children and adolescents. In summary, it has been identified that 12 weeks of multiprofessional interventions are insufficient to reduce WC and AC. These responses can be interrelated with a higher deposition of fat mass in WC compared to NC and AC. Thereby, NC and AC can be used to verify the applicability of these measures in longer-term obesity-treatment interventions.

Regarding the variables of health-related physical fitness, significant improvements were observed in MIHS, MILTS, MILBT, and VO_2_max in the two experimental groups, results that were expected. A relevant point highlighted by Nardo Júnior et al. (2018) discuss that success criteria for the multiprofessional treatment of obesity. The physical fitness variable selected in the study as substantial was aerobic fitness. Improvement in cardiorespiratory fitness reduces risks associated with morbidity and mortality and is considered a key component of the cardiovascular health of adolescents (Nardo Júnior et al., 2018).

Fasting glycemia did not show significant changes in either experimental group after the intervention period. However, the values found in the adolescents before and after the respective time were well below (from 81.7 to 82.6 mg/dL) the cutoff points proposed by the American Diabetes Association, i.e., < 100 mg/dL after fasting for 8 h (American Diabetes Association [ADA], 2018). Nevertheless, significant reductions were observed in the fasting insulin levels in both experimental groups. Thus, it was verified that physical exercise activates the insulin-signaling pathways, facilitating the process of glucose diffusion via GLUT-4 (Röhling et al., 2016). Therefore, considering the mechanisms occurring via GLUT-4, by performing exercise, sensitivity to insulin action is increased and lower levels of insulin release are required for glucose uptake. In regard to lipid profiles, significant reductions in TG, total cholesterol, and LDL-c levels and an elevation in HDL-c were identified in both experimental groups. A systematic review and meta-analysis published by García-Hermoso et al. (2018) identified that concurrent training tends to be more effective for the treatment of variables associated with the lipid profile, particularly in pediatric obesity. A substantial difference was observed for TG levels in this study, and decreases in TG are closely linked to reductions in cardiovascular diseases (Keech and Jenkins, 2017). TG decreases may be associated with PE and suggest changes in feeding during the interdisciplinary intervention (Hartley et al., 2016; Surampudi et al., 2016); such dietary changes may be related to the increased consumption of fibers and omega-3 (Surampudi et al., 2016; Siscovick et al., 2017). However, this is only a hypothesis since feeding control was not conducted during the 12-week interdisciplinary interventions. Considering that, in the present study, the authors did not control food intake over the course of the intervention, it is not possible to confirm that one model is better than the other to further reduce TG levels. Therefore, the differences between the groups can be attributed to a spurious relationship because other variables can influence the responses that are interrelated. Acute studies have identified similar global responses between the order of exercise during concurrent training (Coffey and Hawley, 2017). Nevertheless, in accordance with the author’s knowledge, these are still incipient studies that tested the responses among these biochemical and hormonal variables in order to verify the effectiveness of the order of the concurrent training on these variables. In this way, new longitudinal studies that include food control can be tested to verify the responses between the order of exercise.

Finally, considering that the two models of intervention resulted in similar responses, the choice of the order may be suggested by adolescents in order to maintain adherence, enjoyment, and satisfaction and to reduce dropouts. In addition, another point that can be highlighted is the low cost of the materials needed to conduct the PE routine and to control volume, intensity, and recovery. In light of these, this methodology may be replicated in larger studies in different contexts, e.g., activities against the school shift, sports centers, clubs, and basic health units, among others. Accordingly, this methodology may represent a strategy for health promotion that costs less, considering that the materials are cost effective.

Two limitations may be highlighted: (a) the lack of controlled feeding during the 12-week intervention, and (b) the utilization of perceptual effort and recovery scales in order to quantify training load and recovery, respectively. Despite this, Haddad et al. (2017) suggested that RPE presents good reliability for monitoring training load in teenagers. The same approach was used by Branco et al. (2018) with the purpose of monitoring the training load with PE sessions. Thus, it is believed that familiarization can be incorporated to minimize eventual errors in the utilization of perceptual scales.

## Conclusion

Based on the results presented, the two models of concurrent training within a multiprofessional context focusing on the treatment of obesity positively impacted the body composition and anthropometric variables, reduced FM, %BF, and WC, and elevated MME and RMR. Moreover, regardless of the order in which the concurrent exercise was performed, physical fitness related to health presented significant improvements in maximum isometric strength and cardiopulmonary fitness tests. Finally, in regard to glycemic and lipid profiles, reductions in insulin, HOMA-IR, triglycerides, total cholesterol, and LDL-c and increases in HDL-c were observed in the two experimental groups.

## Data Availability

The datasets generated for this study are available on request to the corresponding author.

## Ethics Statement

This study was approved by the Committee of Ethics in Research of the University Center of Maringá (UniCesumar) through the opinion n 2,505,200/2018.

## Author Contributions

BB, DV, IC, LdO, and SB contributed to conception and design of the study. BB, FdO, DM, and AC organized the database. BB performed the statistical analysis. BB, DV, FdO, IC, DM, AC, LdO, and SB wrote the first draft of the manuscript. BB, IC, LdO, and SB wrote the sections of the manuscript. All authors contributed to the manuscript revision, and read and approved the submitted version.

## Conflict of Interest Statement

The authors declare that the research was conducted in the absence of any commercial or financial relationships that could be construed as a potential conflict of interest.
